# Optimal geometry for a quartz multipurpose SPM sensor

**DOI:** 10.3762/bjnano.4.43

**Published:** 2013-06-17

**Authors:** Julian Stirling

**Affiliations:** 1 School of Physics and Astronomy, The University of Nottingham, University Park, Nottingham, NG7 2RD, United Kingdom

**Keywords:** atomic force microscopy, lateral force microscopy, lateral forces, mechanical vibrations, scanning probe microscopy, scanning tunnelling microscopy

## Abstract

We propose a geometry for a piezoelectric SPM sensor that can be used for combined AFM/LFM/STM. The sensor utilises symmetry to provide a lateral mode without the need to excite torsional modes. The symmetry allows normal and lateral motion to be completely isolated, even when introducing large tips to tune the dynamic properties to optimal values.

## Introduction

The heart of any scanning probe microscope (SPM) is its sensory probe. For a scanning tunnelling microscope (STM) this is simply an electrically conducting wire with an atomically sharp apex. For atomic force microscopes (AFM) and lateral force microscopes (LFM), however, the sensor is more complex. The atomically sharp probe must be combined with a force sensor, usually a cantilever, with either piezoelectric or optical deflection detection. For noncontact AFM (NC-AFM) and dynamic LFM (DLFM), where the sensor is excited at or near one of its eigenfrequencies, properties such as the Q factor, eigenfrequencies, effective spring constant [1] and other geometrical properties [2] of the eigenmodes become important.

AFM and LFM sensors have evolved from gold foil with diamond tip [3] and bent tungsten wires [4] respectively, into a wide range of specialised sensors. The most common NC-AFM sensors: silicon microcantilvers [5], and quartz sensors such as the qPlus sensor (tuning fork) [6] or KolibriSensor^®^ [7], have all been used for combined AFM/STM [7–9]. Combined AFM/LFM sensors have been constructed from silicon cantilevers, by exciting torsional modes to generate the lateral motion needed for the LFM [10]. The qPlus sensor has been used as an LFM by rotating the tip on the end of the quartz tuning fork [11], but no combined AFM/LFM qPlus system has been developed due to the magnitude of the torsion constant for the tine of the sensor. A combined AFM/LFM sensor operated in frequency modulation mode would enable measurements of conservative and nonconservative forces simultaneously in the normal and lateral direction. Such measurements could be used to further important investigations in single-asperity friction [12], where the relationship between normal and lateral force is of interest. In this paper, we suggest the optimum geometry of a quartz sensor to produce a combined AFM/LFM/STM from a quartz crystal resonator with many theoretical benefits over other sensors.

### Combining NC-AFM and DLFM

For a sensor to image as both an NC-AFM and a DLFM the sensor must be able to oscillate both normal to and parallel to the surface it is scanning. The simplest method for achieving this is a single oscillator which will oscillate in different directions depending on the eigenmode excited. Ideally for atomic-resolution imaging the effective spring constant of the excited eigenmode should be low [13]. However, as the spring constant normal to the surface lowers, the risk of the probe snapping to contact with the surface increases. This produces a problem for combined AFM/LFM using the principal and first torsional eigenmode of a cantilever, as the torsional mode can have an effective spring constant of up to approximately two orders of magnitude higher than the principal mode [10]. This results in a difficult tradeoff. To avoid snap to contact, the following condition must be satisfied [14]:

[1]
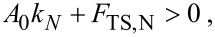


where *A*_0_ and *k_N_* are the amplitude and effective spring constant of the principal eigenmode, and *F*_TS,N_ is the tip–sample force normal to the sample.

For high-resolution AFM imaging *A*_0_ should be as low as possible [13]. However, the signal-to-noise ratio, which is a function of *A*_0_, [15] limits the minimum amplitude. Experimentally, some groups have achieved stable imaging with amplitudes as low as 20 pm [16]. Thus, to be safe from snap to contact for atomic forces on the order of −3 nN, it is required that *k_N_* > 150 N·m^−1^. If imaging, however, is only in DLFM mode, then *A*_0_ is ideally zero. Obviously, Equation 1 doesn’t hold in this case as it would suggest that we require an infinite spring constant to stop snap to contact. In this example *A*_0_ must be considered as the distance the tip has moved from its equilibrium position due to *F*_TS,N_. Therefore, if trying to image in DLFM mode, the error in the *z*-position due to normal forces is inversely proportional to *k_N_*, requiring higher minimum normal spring constants of *k_N_* ≥ 1–3 kN·m^−1^. This would result in torsional constants on the order of hundreds of kN·m^−1^, which is not ideal for LFM imaging.

The torque required to torsionally twist a beam of length *L* through an angle θ is given by

[2]
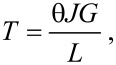


where *J* and *G* are the torsion constant and shear modulus of the beam. In the case of a cantilever beam with a tip of length *L*_tip_ (measured from the central axis of the beam), the lateral displacement of the tip apex, *A*_lat_, is *L*_tip_θ. Replacing the torque with the lateral tip–sample force *F*_TS,L_ multiplied by the tip length we get

[3]
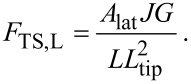


Hence, the lateral spring constant

[4]
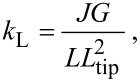


is inversely proportional to the square of the tip length. Thus, the tip length becomes an important parameter to consider alongside the more typical geometrical constants associated with the normal spring constant.

For quartz sensors the obvious choice of cantilever is the standard qPlus sensor with a normal spring constant of approximately 1.8 kN·m^−1^. [17–18] For commercially available silicon cantilevers the spring constants are usually less than 50 N·m^−1^, with resonant frequencies of 200–300 kHz. The resonant frequency of the cantilever scales with *L*^−4^ and the spring constant with *L*^−3^. Considering that the torsional eigenfrequency can be approximately two orders of magnitude larger than the normal eigenfrequency, achieving the necessary normal spring constant by length reduction could push the torsional eigenfrequency into the gigahertz range, which is impractical. We instead will consider different rectangular cross sections for a 200 μm long silicon beam.

A range of rectangular cross sections which would produce a normal spring constant of 2 kN·m^−1^ for a 200 μm long beam have been calculated, using the Euler–Bernoulli beam theory, see Table 1. This value was chosen to sit in the middle of the range suggested for the minimum normal spring constant. The frequency of the first eigenmode has also been calculated. Using Equation 4, the tip length needed for *k*_L_ = 2 kN·m^−1^ was calculated, using previously tabulated values for *J* [19]. This tip length was also calculated for the qPlus sensor.

**Table 1:** Dimensions and dynamic properties of silicon microcantilevers that would provide normal spring constants of 2 kN·m^−1^. *L*_tip_ is the tip length required to provide a lateral spring constant of 2 kN·m^−1^. qPlus sensor is also included (the frequency is not 32768 Hz, as some features, such as base deformations and gold contacts, were neglected in the calculations).

Name	*L* (μm)	*w* (μm)	*T* (μm)	*k_N_* (N·m^−1^)	*f_N_* (Hz)	*L*_tip_ (μm)	*L*_tip_/*L*

Si1	200	112.2	15.0	2 000	515 913	139.1	0.696
Si2	200	47.3	20.0	2 000	687 884	124.6	0.623
Si3	200	24.2	25.0	2 000	859 856	93.1	0.466
Si4	200	14.0	30.0	2 000	1 031 827	57.2	0.286
Si5	200	8.8	35.0	2 000	1 203 798	33.7	0.168
Si6	200	5.9	40.0	2 000	1 375 769	20.5	0.102
qPlus	2 400	130.0	214.0	1 763	32 246	772.9	0.322

The calculated tip lengths range from approximately 10% to 70% of the beam length. As has previously been shown, AFM sensors with tip lengths of similar scale to the length of the beam exhibit a large lateral component to the motion of the tip apex in the first eigenmode [2]. This lateral component is perpendicular to the torsional eigenmode, thus making it impossible to truly separate the normal and lateral forces. This problem is exacerbated if the tip length is further increased to increase sensitivity to lateral forces by reducing the lateral spring constant, as snap to contact is not an issue in the lateral direction. Increasing the ratio of thickness to width reduces the required tip length, but at the expense of introducing normal eigenfrequencies above 1 MHz, pushing torsional eigenfrequencies to ranges that most AFM electronics cannot handle.

### Non-cantilever geometries

**Figure 1:**
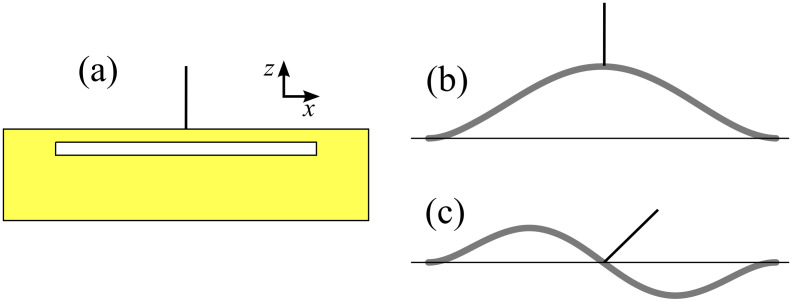
(a) Proposed geometry of new sensor. A tungsten tip connected to the centre of a quartz bar clamped at both ends. (b) First and (c) second eigenmode of the sensor. The symmetry provides pure normal motion in the first eigenmode and lateral motion of the tip apex in the second eigenmode due to the rotation of the tip about the antinode of the beam.

Due to the large difference between the operating frequencies of normal and torsional modes, and the coupling of unwanted lateral motion into the normal eigenmode for sensors with the tip lengths needed to produce low lateral spring constants, we propose a new sensor geometry. The proposed design, see Figure 1, is to attach a tungsten tip to the centre of a quartz beam. The design exploits the intrinsic symmetry of the sensor to remove any unwanted lateral motion in the principal eigenmode (Figure 1b), thus allowing for longer tips. By exciting the second eigenmode of the beam, lateral motion can be generated (Figure 1c). The effective spring constant and eigenfrequency can be calculated, and thus tuned, far more simply than for torsional modes, by solving the Euler–Bernoulli beam equation with the appropriate boundary conditions. In principle, by also exciting a torsional mode, a perpendicular lateral oscillation could be generated allowing simultaneous measurements in *all three dimensions*. This paper will, however, concentrate on just the first and second eigenmode.

## Results and Discussion

### Dynamic properties of symmetric sensors

#### Spring constants

The two most fundamental properties to consider for dynamic force sensors are the effective spring constants and the eigenfrequencies of each imaging mode. For simplicity we will begin with effective spring constants, as the influence of the inertia of the tip has only the effect of moving the dynamic spring constant closer to the static constant [20], removing the ≈3% error. (Note that this is not true in higher eigenmodes for cantilever geometries as the inertia shifts the position of the antinodes [1]. In this system, however, the antinodes are pinned due to the symmetry of the system.)

The dynamic Euler–Bernoulli beam equation

[5]
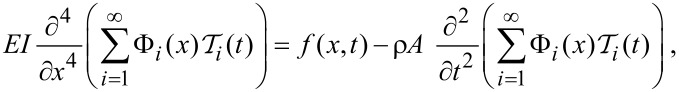


describes the dynamic deformations of a beam, where *E* and *ρ* are the Young’s modulus and density of the material, respectively. *A* and *I* are the area and second moment of area of the cross section of the beam. *f*(*x*,*t*) is the applied force per unit length acting on the beam, Φ*_i_*(*x*) and 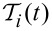 are the spatial and temporal components of the beam’s deformation for the *i*th eigenmode.

As any effect from the tip must be considered at the centre of the beam we will consider only one half of the beam and use symmetry (or antisymmetry in the case of even eigenmodes) to construct the full spatial solution. For both even and odd modes the boundary conditions

[6]
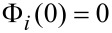


[7]
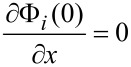


are valid. Equation 5 is spatially fourth order, therefore two further conditions are required. For odd modes:

[8]
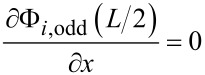


[9]
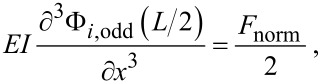


and for even modes:

[10]
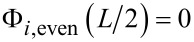


[11]
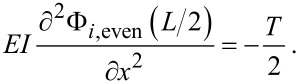


*L* is the length of the entire beam, and *F*_norm_ and *T* are the force and torque applied to the centre of the beam.

Entering these boundary conditions into the general static spatial solution of Equation 5 (i.e., the final term is zero), gives the spring constant of the first eigenmode as

[12]
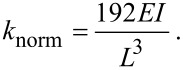


Considering the torque on the centre of the beam *T* = *L*_tip_*F*_lat_, where *F*_lat_ is a lateral force applied in the *x* direction at the far end of the tip, the effective lateral spring constant of the second eigenmode is

[13]
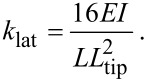


Full derivations are provided in Supporting Information File 1.

Thus, just as in the case of the torsional mode, the effective spring constant in the lateral mode can be tuned by tip length. However, due to the symmetry of the sensor this will not cause unwanted lateral motion at the tip apex in the first eigenmode.

#### Eigenfrequencies

When considering the eigenfrequencies of the sensor, the inertia of the tip plays a very strong role, which cannot be ignored. Solving Equation 5 for the dynamic case, the same boundary conditions (Equation 6–Equation 11) hold, where

[14]
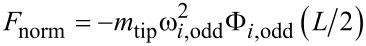


and

[15]
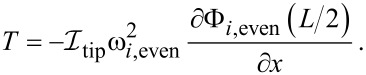


*ω_i_* is the angular eigenfrequency of the *i*th eigenmode, and 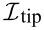 is the moment of inertia of the tip. By combining the general spatial solution with the four boundary conditions as a matrix equation, equal to a zero vector, we see that resonance occurs when the determinant is equal to zero (See Supporting Information File 1 for full derivation). Giving the following resonance conditions:

[16]
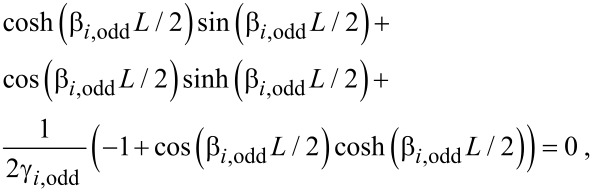


and

[17]
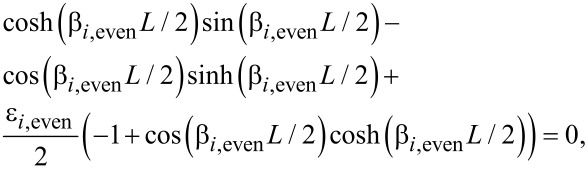


where

[18]
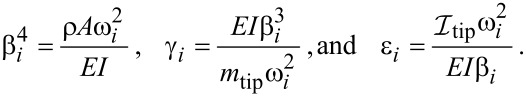


These equations can be solved numerically in terms of dimensionless quantities (*β_i_L*, m^*^, 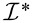, discussed in Supporting Information File 1), and dimensions can be added later to get a value for ω_i_. In the case of no tip, the ratio between the second and first eigenmode is 2.757. Whether this ratio rises or falls when a tip is added depends on the dimensions of both the tip and sensor. It is clear, however, that such a low ratio between the eigenmodes is another advantage of the symmetric sensor over torsional designs as both modes can be tuned to near the optimal frequency of the detection system.

### Optimal geometry for a symmetric sensor

In order for the sensor to be used in currently available commercial UHV combined AFM/STM systems, it should be similar in size to the qPlus sensor. However, as the normal spring constant per unit beam length (with the same cross section) is 64 times higher than for a cantilever geometry, a greater length than the 2.4 mm beam of the qPlus sensor is advisable.

Choosing a 3 mm long beam and a normal spring constant of 2 kN·m^−1^, as previously suggested, we calculate that the second moment of area of the cross section should be *I* = 3.68 × 10^−18^ m^4^. A width (y-direction) *w* of 100 μm would result in a thickness (z-direction) of *t* = 76.1 μm, as *I* = *wt*^3^/12. Such a beam would have first and second eigenfrequencies of 46.7 kHz and 128.8 kHz, respectively. These frequencies will reduce when the tip is added to the centre of the beam.

Before considering the mass or moment of inertia of the tip and its effect on the eigenfrequencies of the sensor, it is important to consider the spring constant of the tip itself. Any bending of the tip will not be detected by the piezoelectric quartz sensor. Thus, treating the tip as a cantilever, its spring constant must be much greater than the effective lateral spring constant for the sensor (*k*_lat_), otherwise this will result in incorrect force measurements in the LFM mode. We consider a maximum tip length of 1.73 mm, i.e., the length that would give *k*_lat_ = 500 N·m^−1^; thus, to keep the spring constant of the tip above 10 kN·m^−1^ the diameter of the tungsten wire must be greater than 144 μm.

**Figure 2:**
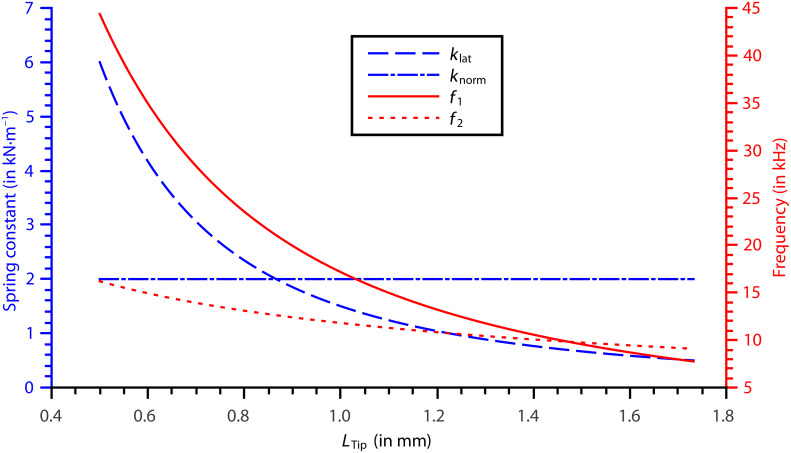
The effective spring constants (*k*_lat_ for mode 2, and *k*_norm_ for mode 1) and eigenfrequencies of the first two eigenmodes of a symmetric sensor. Plotted for 150 μm tungsten tips of varying lengths.

We will consider a tip diameter, *D*_tip_, of 150 μm, an easily available diameter of tungsten wire. The moment of inertia of the tip for the even modes should be calculated about the centre of the beam, *t*/2 from the bottom of the tip, and hence a distance of (*L*_tip_ + *t*)/2 from the centre of mass of the tip. Thus, the moment of inertia of the tip can simply be calculated by the parallel axis theorem as 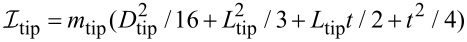. By using Equation 13 and Equation 16–Equation 18, the spring constants and eigenfrequencies of the first two modes have been plotted in Figure 2 for a range of tip lengths. For plotted tip lengths the ratio of the spring constant of the tip to *k*_lat_ is at its minimum 23.5.

Examining the plot it is clear that tip lengths near 1.47 mm are unusable as the two eigenfrequencies are too close. This would make it difficult to selectively excite them, as well as require long averaging times in bimodal operation to remove any correlation between the modes. The benefit of increasing the tip length is a reduction in lateral spring constant, which comes at the price of lower eigenfrequencies. A tip length of 1mm would provide eigenfrequencies of *f*_1_ = 11.8 kHz and *f*_2_ = 17.2 kHz, with *k*_lat_= 1.50 kN·m^−1^. These frequencies are of the same order of magnitude as qPlus sensors with long tips, thus the sensor could be used in commercially available qPlus systems with no modifications to the electronics.

It is also important to consider the minimum amplitudes achievable by the sensor, particularly in the lateral mode. As little is know about the optimum amplitudes in DLFM, this issue is to be treated approximately. The lateral amplitude of the tip apex is

[19]
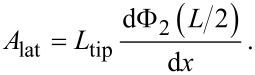


By considering that the tip is approximately half the length of the beam (*L*/2), and (dΦ_2_(*L*/2))/d*x* ≈ (4*A*_antinode_)/*L* (approximating Figure 1c as three straight lines), where *A*_antinode_ is the amplitude of the antinode, the ratio between *A*_lat_ and *A*_antinode_ is on the order of 2. Other detection parameters are also of the same order of magnitude as for a qPlus sensor. Thus, as qPlus sensors have achieved imaging with amplitudes as low as 20 pm [16], similar amplitudes are in theory possible for the LFM mode of the symmetric sensor. Such amplitudes are an order of magnitude smaller than inter-atomic distances.

### Experimental viability

The experimental viability of this method depends on the equipment available to produce the sensor. Firstly, no quartz crystal resonators of the proposed geometry are commercially available. The closest commercially available sensor is a double-ended tuning fork available from Statek (DETF Force Sensor, http://www.statek.com). By fixing the bottom tine it is possible to reproduce the required symmetry. However, these sensors are too large for most commercial qPlus systems with a total width of 15.2 mm and a beam length of 8.44 mm. Also the beams are recessed with respect to the top of the resonator by 0.86 mm, preventing tip lengths below this. A second possible option would be to attach two identical tuning forks end-to-end by using a similar method to Heyde et al. [21]; however, the glue used to attach the tines will have different mechanical properties to the quartz and also depend on the quantity, placement, and curing conditions. This will affect the repeatability as well as the shape of the eigenmodes, and hence the spring constant. Thus, ideally custom resonators would need to be made. Secondly, the correct placement of the tungsten wire is vital. The tip needs to be positioned in the centre of the 3 mm beam, which is just 100 × 76.1 μm in cross section, and needs to be mounted perfectly normal to the beam. Misplacement of the tip breaks the symmetry thus affecting operation. Reproducible tip placement requires three dimensional micromanipulators, which can be prohibitively expensive for some groups.

A final consideration should be taken regarding the connection of a separate electrode for the tunnel current. Two options are available, first a thin (about 15–50 μm) loose wire could be attached to the tungsten tip, as is often done for qPlus sensors. This is inadvisable as it also breaks the symmetry of the sensor. Another method would be to add a thin insulating layer to the top side of the resonator and on top of that a new electrode, such as the method developed by Nauga Needles [22]. This maintains the symmetry; however, great care needs to be taken to consider the possibility of capacitive cross-talk between the tunnelling and deflection channels [23].

## Conclusion

We have demonstrated a new geometry of a piezoelectric sensor for use in combined AFM/LFM, which utilises symmetry to bring the eigenfrequencies and spring constants of the two modes closer together. This allows both modes be tuned to the optimal parameters for operation. The symmetry also removes issues with unwanted lateral motion in normal oscillating modes, allowing longer tips for tuning the lateral spring constant of the LFM operation. By attaching an extra electrode, the sensor can also be used for STM, providing a truly multipurpose SPM sensor.

## Supporting Information

File 1 Full derivations of dynamic properties for a symmetric sensor
