# Supplementary material for: Optimal geometry for a quartz multipurpose SPM sensor
Source: Beilstein J Nanotechnol. 2013 Jun 17;4:370–6. doi: 10.3762/bjnano.4.43 (PMC3701426; doi:10.3762/bjnano.4.43)
Supplement: File 1 — Full derivations of dynamic properties for a symmetric sensor [file Beilstein_J_Nanotechnol-04-370-s001.pdf]

Supporting Information

for

Optimal geometry for a quartz multipurpose SPM sensor

Julian Stirling*

Address: School of Physics and Astronomy, The University of Nottingham, University Park, Nottingham, NG7 2RD, United Kingdom

Email: Julian Stirling - ppxjs1@nottingham.ac.uk

* Corresponding author

Full derivations of dynamic properties for a symmetric sensor

Harmonics of the symmetric sensor

Considering the boundary conditions for a symmetric sensor is a little more difficult than for a cantilever, as once the effects of the tip or interactions are included they will occur at the centre of the beam. The best method to account for this is to consider that, for odd modes, the cantilever will be symmetric, and for even modes antisymmetric. Now we can consider the boundary conditions at one of the clamped ends and at the centre of the beam.

Odd modes

Neglecting tip mass for simplicity, the boundary conditions for the odd modes are

$$\Phi_i(0) = 0 \tag{1}$$

$$\frac{\mathrm{d}\Phi_i(0)}{\mathrm{d}x} = 0 \tag{2}$$

$$\frac{\mathrm{d}\Phi_i(L/2)}{\mathrm{d}x} = 0 \tag{3}$$

$$\frac{\mathrm{d}^3\Phi_i(L/2)}{\mathrm{d}x^3} = 0 \tag{4}$$

Thus, considering the normalised spatial solution as

$$\Phi_i(x) = b_1 \cos(\beta_i x) + b_2 \sin(\beta_i x) + b_3 \cosh(\beta_i x) + b_4 \sinh(\beta_i x)\,, \tag{5}$$

where

$$\beta_i^4 = \frac{\rho A \omega_i^2}{EI}\,. \tag{6}$$

This can be written as the following matrix equation

$$\underline{\underline{D}} \begin{pmatrix} b_1 \\ b_2 \\ b_3 \\ b_4 \end{pmatrix} = \begin{pmatrix} 0 \\ 0 \\ 0 \\ 0 \end{pmatrix}, \tag{7}$$

where

$$\underline{\underline{D}} = \begin{pmatrix} 1 & 0 & 1 & 0 \\ 0 & 1 & 0 & 1 \\ -\sin(\beta_i L/2) & \cos(\beta_i L/2) & \sinh(\beta_i L/2) & \cosh(\beta_i L/2) \\ \sin(\beta_i L/2) & -\cos(\beta_i L/2) & \sinh(\beta_i L/2) & \cosh(\beta_i L/2) \end{pmatrix}. \tag{8}$$

Now for a nontrivial solution $\det \underline{\underline{D}} = 0$. This gives the resonance conditions

$$\cosh(\beta_i L/2)\sin(\beta_i L/2) + \cos(\beta_i L/2)\sinh(\beta_i L/2) = 0 \tag{9}$$

From equations 1 and 2 we can see that

$$b_1 = -b_3 \qquad\qquad b_2 = -b_4 \tag{10}$$

and from Equation 3 we can get the ratio between the constants:

$$\frac{b_1}{b_2} = \frac{\cos(\beta_i L/2) - \cosh(\beta_i L/2)}{\sin(\beta_i L/2) + \sinh(\beta_i L/2)} \tag{11}$$

Even modes

For the even modes the boundary conditions are slightly different

$$\Phi_i(0) = 0 \tag{12}$$

$$\frac{\mathrm{d}\Phi_i(0)}{\mathrm{d}x} = 0 \tag{13}$$

$$\Phi_i(L/2) = 0 \tag{14}$$

$$\frac{\mathrm{d}^2\Phi_i(L/2)}{\mathrm{d}x^2} = 0 \tag{15}$$

thus for these cases

$$\underline{\underline{D}} = \begin{pmatrix} 1 & 0 & 1 & 0 \\ 0 & 1 & 0 & 1 \\ \cos(\beta_i L/2) & \sin(\beta_i L/2) & \cosh(\beta_i L/2) & \sinh(\beta_i L/2) \\ -\cos(\beta_i L/2) & -\sin(\beta_i L/2) & \cosh(\beta_i L/2) & \sinh(\beta_i L/2) \end{pmatrix}. \tag{16}$$

giving a condition for resonance of

$$\cosh(\beta_i L/2)\sin(\beta_i L/2) - \cos(\beta_i L/2)\sinh(\beta_i L/2) = 0 \tag{17}$$

As before, from equations 12 and 13 we can see that

$$b_1 = -b_3 \qquad\qquad b_2 = -b_4 \tag{18}$$

but Equation 14 gives a slightly different ratio between the constants:

$$\frac{b_1}{b_2} = \frac{\sinh(\beta_i L/2) - \sin(\beta_i L/2)}{\cos(\beta_i L/2) - \cosh(\beta_i L/2)} \tag{19}$$

Odd modes - including tip

Including the mass of the tip requires a simple change of the 4th boundary condition to

$$EI\frac{\mathrm{d}^3\Phi_i(L/2)}{\mathrm{d}x^3} = -\frac{m_{\mathrm{tip}}}{2}\omega_i^2\Phi_i(L/2) \tag{20}$$

Again, writing this as a matrix equation,

$$\underline{\underline{D}}\begin{pmatrix} b_1 \\ b_2 \\ b_3 \\ b_4 \end{pmatrix} = \begin{pmatrix} 0 \\ 0 \\ 0 \\ 0 \end{pmatrix}, \tag{21}$$

where

$$\underline{\underline{D}} = \begin{pmatrix} 1 & 0 & \cdots \\ 0 & 1 & \\ -\sin(\beta_i L/2) & \cos(\beta_i L/2) & \\ \sin(\beta_i L/2) + \frac{1}{2\gamma_i}\cos(\beta_i L/2) & -\cos(\beta_i L/2) + \frac{1}{2\gamma_i}\sin(\beta_i L/2) & \end{pmatrix}$$

$$\begin{matrix} & 1 & 0 \\ & 0 & 1 \\ \cdots & \sinh(\beta_i L/2) & \cosh(\beta_i L/2) \\ & \sinh(\beta_i L/2) + \frac{1}{2\gamma_i}\cosh(\beta_i L/2) & \cosh(\beta_i L/2) + \frac{1}{2\gamma_i}\sinh(\beta_i L/2) \end{matrix}\Bigg) \tag{22}$$

where

$$\gamma_i = \frac{EI\beta_i^3}{m_{\mathrm{tip}}\omega_i^2} \tag{23}$$

Now for a nontrivial solution $\det \underline{\underline{D}} = 0$. This gives the resonance conditions

$$\cosh(\beta_i L/2)\sin(\beta_i L/2) + \cos(\beta_i L/2)\sinh(\beta_i L/2) + \frac{1}{2\gamma_i}\big(-1 + \cos(\beta_i L/2)\cosh(\beta_i L/2)\big) = 0\,. \tag{24}$$

To calculate the frequencies, we must first combine Equation 23 with Equation 6 to remove the ω_i dependence in the definition of γ_i:

$$\gamma_i = \frac{m_\mathrm{b}}{m_\mathrm{tip}\beta_i L} = \frac{1}{m^*\beta_i L}, \tag{25}$$

where m_b is the mass of the beam, and m^* is the ratio of the tip mass to the beam mass. Using this form of γ_i in Equation 24 allows the the dimensionless quantity $\beta_i L$ to be solved for any m^* by a simple numerical method such as Newton-Raphson. Dimensions can be subsequently added to calculate ω_i.

As equations 1, 2 and 3 are still valid, the ratio between the constants remains as

$$\frac{b_1}{b_2} = \frac{\cos(\beta_i L/2) - \cosh(\beta_i L/2)}{\sin(\beta_i L/2) + \sinh(\beta_i L/2)} \tag{26}$$

Even modes - including tip

For the even modes the first three boundary conditions are also unaffected by the tip, and the fourth changes to

$$EI\frac{\mathrm{d}^2\Phi_i(L/2)}{\mathrm{d}x^2} = \frac{\mathscr{I}_\mathrm{tip}}{2}\omega_i^2\frac{\mathrm{d}\Phi_i(L/2)}{\mathrm{d}x}, \tag{27}$$

where $\mathscr{I}_\mathrm{tip}$ is the moment of inertia of the tip.

Writing the four new conditions as a matrix

$$\underline{\underline{D}} = \begin{pmatrix} 1 & 0 & 1 & 0 \\ 0 & 1 & 0 & 1 \\ \cos(\beta_i L/2) & \sin(\beta_i L/2) & \cosh(\beta_i L/2) & \sinh(\beta_i L/2) \\ -\cos(\beta_i L/2) + \frac{\varepsilon_i}{2}\sin(\beta_i L/2) & -\sin(\beta_i L/2) - \frac{\varepsilon_i}{2}\cos(\beta_i L/2) & \cosh(\beta_i L/2) - \frac{\varepsilon_i}{2}\sinh(\beta_i L/2) & \sinh(\beta_i L/2) - \frac{\varepsilon_i}{2}\cosh(\beta_i L/2) \end{pmatrix}, \tag{28}$$

where

$$\varepsilon_i = \frac{\mathscr{I}_{\text{tip}}\omega_i^2}{EI\beta_i} \tag{29}$$

This gives a resonance condition of

$$\cosh(\beta_i L/2)\sin(\beta_i L/2) - \cos(\beta_i L/2)\sinh(\beta_i L/2) + \frac{\varepsilon_i}{2}\big(-1 + \cos(\beta_i L/2)\cosh(\beta_i L/2)\big) = 0\,. \tag{30}$$

To calculate the frequencies we must first combine Equation 29 with Equation 6 to remove the ω_i dependence in the definition of ε_i:

$$\varepsilon_i = \frac{\mathscr{I}_{\text{tip}}(\beta_i L)^3}{L^2 m_{\text{b}}} = \mathscr{I}^*(\beta_i L)^3\,, \tag{31}$$

where $\mathscr{I}^*$ is the ratio of the moment of inertia of the tip to the beam mass multiplied by the beams length squared. This ratio, while physically meaningless, as $m_{\text{b}}L^2$ is not the moment of inertia of the beam for any relevant rotational axis, provides a dimensionless constant to solve Equation 30.

Again $\beta_i L$ can be solved for any $\mathscr{I}^*$ by the Newton–Raphson method and dimensions can be added to calculate ω_i.

Again as equations 12, 13 and 14 are still valid the ratio between the constants remains as

$$\frac{b_1}{b_2} = \frac{\sinh(\beta_i L/2) - \sin(\beta_i L/2)}{\cos(\beta_i L/2) - \cosh(\beta_i L/2)} \tag{32}$$

Static Spring Constants

Static spring constants are not normally (by definition) linked to the modes. However, they are linked to the symmetry and boundary conditions. Thus, the even and odd modes relate to two separate static spring constants. One for the end of the tip being pushed normal to the beam (k_{norm}), and the other for the tip being pushed parallel to the beam (k_{lat}).

Calculating k_{norm}

In the normal case our boundary conditions are now

$$\Phi_i(0) = 0 \tag{33}$$

$$\frac{\mathrm{d}\Phi_i(0)}{\mathrm{d}x} = 0 \tag{34}$$

$$\frac{\mathrm{d}\Phi_i(L/2)}{\mathrm{d}x} = 0 \tag{35}$$

$$EI\frac{\mathrm{d}^3\Phi_i(L/2)}{\mathrm{d}x^3} = \frac{F_{\text{norm}}}{2} \tag{36}$$

where F_{norm} is the force on the tip normal to the beam. The factor of two is because we are only considering the force felt by half of the beam.

The general static spatial solution is

$$\Phi_i = A + Bx + Cx^2 + Dx^3 \tag{37}$$

from Equation 33 and Equation 34 we get

$$A = 0 \qquad \text{and} \qquad B = 0\,. \tag{38}$$

With Equation 35 we get

$$C = -\frac{3DL}{4} \tag{39}$$

and finally using Equation 36 we can show

$$D = \frac{F_{\text{norm}}}{12EI} \tag{40}$$

combining this with Equation 39

$$C = \frac{-F_{\text{norm}}L}{16EI} \tag{41}$$

Therefore giving the solution of x for these boundary conditions as

$$z = \frac{F_{\text{norm}}}{4EI}\left(\frac{1}{3}x^3 - \frac{1}{4}Lx^2\right)\,. \tag{42}$$

Considering the deflection a $x = L/2$

$$F_{\text{norm}} = \frac{-192EI}{L^3}A_{\text{norm}} \tag{43}$$

Therefore using Hooke's law, the static spring constant of the normal mode of a double-ended tuning-fork sensor is

$$k_{\text{norm}} = \frac{192EI}{L^3}\,. \tag{44}$$

Calculating k_{lat}

In the lateral case our boundary conditions are now

$$\Phi_i(0) = 0 \tag{45}$$

$$\frac{\mathrm{d}\Phi_i(0)}{\mathrm{d}x} = 0 \tag{46}$$

$$\Phi_i(L/2) = 0 \tag{47}$$

$$EI\frac{\mathrm{d}^2\Phi_i(L/2)}{\mathrm{d}x^2} = -\frac{F_{\text{lat}}H}{2} \tag{48}$$

where F_{lat} is the force on the tip parallel to the beam, and H is the tip length. The factor of two is because we are only considering the force felt by half of the beam.

The general static spatial solution is still

$$\Phi_i = A + Bx + Cx^2 + Dx^3 \tag{49}$$

from Equation 45 and Equation 46 we get

$$A = 0 \qquad \text{and} \qquad B = 0 \tag{50}$$

as before. With Equation 47 we get

$$C = -DL \tag{51}$$

and finally using Equation 48 we can show

$$D = \frac{-F_{\text{lat}}H}{4EIL} \tag{52}$$

combining this with Equation 51

$$C = \frac{F_{\text{lat}} H}{8EI} \tag{53}$$

Therefore giving the solution of x for these boundary conditions as

$$z = \frac{F_{\text{lat}} H}{4EI} \left(\frac{1}{2} x^2 - \frac{1}{L} x^3 \right) . \tag{54}$$

Considering the deflection a $x = L/2$, $z = 0$, as expected. Considering the first derivative of the deflection

$$\frac{\mathrm{d}z}{\mathrm{d}x} = \frac{F_{\text{lat}} H}{4EI} \left(x - \frac{3}{L} x^2 \right) . \tag{55}$$

at $x = L/2$

$$\frac{\mathrm{d}z(L/2)}{\mathrm{d}x} = \frac{F_{\text{lat}} HL}{16EI} . \tag{56}$$

However as $A_{\text{lat}} = -H \frac{\mathrm{d}z(L/2)}{\mathrm{d}x}$

$$F_{\text{lat}} = -\frac{16EI}{H^2 L} A_{\text{lat}} \tag{57}$$

Therefore using Hooke's law, the static spring constant of the lateral mode of a double-ended tuning-fork sensor is

$$k_{\text{lat}} = \frac{16EI}{H^2 L} . \tag{58}$$
